# An Unusual Cause of Respiratory Distress in Term Neonate

**DOI:** 10.7759/cureus.27547

**Published:** 2022-08-01

**Authors:** Jubara S Alallah, Reham Makki, Arwa A Saber, Ahmed Moustafa, Hasan Ghandourah

**Affiliations:** 1 Neonatology, King Abdulaziz Medical City, Jeddah, SAU; 2 Collage of Medicine, King Saud Bin Abdulaziz University for Health Sciences, Jeddah, SAU; 3 Neonatology, King Abdullah International Medical Research Center, Jeddah, SAU; 4 College of Medicine, King Saud Bin Abdulaziz University for Health Sciences, Jeddah, SAU; 5 Pediatrics/Pulmonology, King Abdulaziz Medical City, Jeddah, SAU; 6 Pediatrics, King Abdullah International Medical Research Center, Jeddah, SAU

**Keywords:** rsph9 gene, primary ciliary dyskinesia, genetic testing, respiratory distress, newborn

## Abstract

We report a female infant who was born at 41+6 weeks of gestation to a consanguineous parent, and the initial newborn examination was within normal. At 12 hours of age, she developed tachypnea; with desaturation, she had continuous thick whitish oral secretion. Admitted to the neonatal intensive care unit (NICU) for further management, her initial blood investigation, including blood gas and chest X-ray, was normal. Due to the persistent unexplained respiratory distress with a normal chest X-ray, we obtained a further history from parents with three siblings with respiratory symptoms but no definitive diagnosis. The genetic testing of whole-exome sequences (WES) confirmed a homozygous variant c.804_806del, p.(Lys268del) in the RSPH9 gene that causes primary ciliary dyskinesia (PCD). Her three siblings were tested and found to have the same genetic mutation.

## Introduction

Primary ciliary dyskinesia (PCD) is a rare autosomal recessive genetic disorder that causes defects in the function and/or structure of the cilia lining the respiratory tract, fallopian tube, and flagellum of sperm cells [1]. PCD is often underdiagnosed, and it is estimated to occur in 1/15,000-20,000 individuals [2-3]. Moreover, due to the high consanguinity rates, PCD is more common in Arab societies, although little is known about its actual prevalence and characteristics [4]. Patients with PCD may present with neonatal respiratory distress and/or laterality defects in about half of the cases. Studies have shown that more than 80% of neonate patients present with respiratory distress symptoms within the first 1-2 days of life, with most cases appearing 12 hours after birth [3]. In this article, we report an unusual case of respiratory distress in a full-term female infant. The diagnosis of primary ciliary dyskinesia was confirmed by genetic testing, which led to the same diagnosis in three siblings at different ages. 

## Case presentation

**Figure 1:**
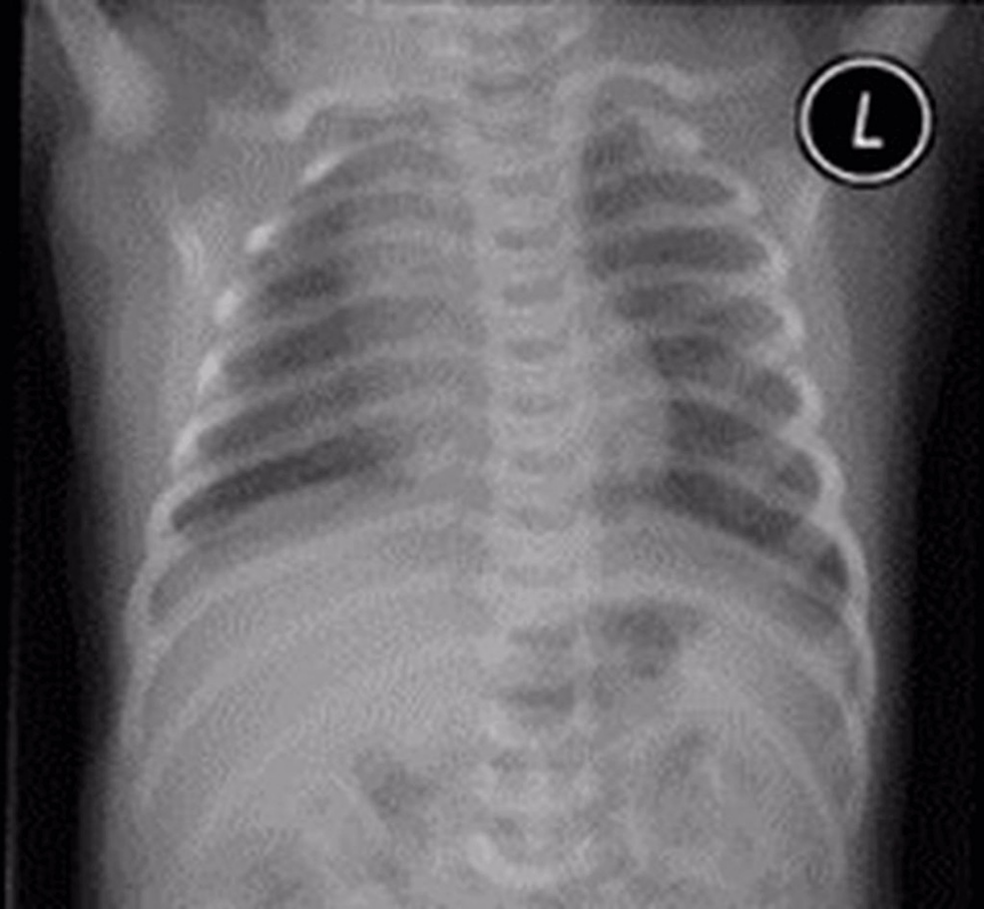
Normal chest X-ray with normal cardiac shadow

A female infant was born at 41+6 weeks of gestation via an emergency Cesarean section due to abnormal fetal heart rhythm. The infant's mother was diabetic. The parent is a first-degree cousin. She had three siblings diagnosed with bronchial asthma and chronic otitis media. Antenatal ultrasounds were unremarkable, and the maternal laboratory findings are as follows: hepatitis B was negative; group B Streptococcus was positive. Her appearance, pulse, grimace, activity, and respiration (APGAR) scores were nine and nine at one and five minutes, respectively, and her weight was 4 kg. Her vital signs were stable (temperature of 36.9ºC; heart rate of 150 beats/min; respiratory rate of 55 breaths/min; blood pressure of 66/40 mmHg; oxygen saturation of 96%), and the initial newborn examination presented normal results. At 12 hours of age, the baby started to be tachypneic; with desaturation in room air, she had continuous thick whitish oral secretions. A full sepsis workup was done. Complete blood cell count revealed a white blood cell count of 6,000/mL with a normal differential, hemoglobin of 15.8 g/dL, and platelet count of 250×10^3^/μL. Her initial blood gas and chest X-ray were normal (Figure 1). 

An echocardiogram showed normal structure and function of the heart with no evidence of pulmonary hypertension. Initially, she was on a high-flow nasal cannula (HFNC) of eight liters per minute with a fraction of inspired oxygen (FiO_2_) of 35%. She was treated for clinical sepsis with five days of antibiotics. Her respiratory distress improved with respiratory support. She needed oxygen for a total period of 14 days, and then she was observed for two days without supplemental oxygen before being discharged. The genetic testing was requested based on unexplained respiratory distress with normal chest X-ray and normal echocardiography. The whole-exome sequences (WES) confirmed a homozygous variant c.804 to 806del, p.(Lys268del) in the RSPH9 gene (OMIM: 612648) that causes PCD. Her three siblings were tested for the same gene and confirmed the same genetic mutation.

**Figure 2:**
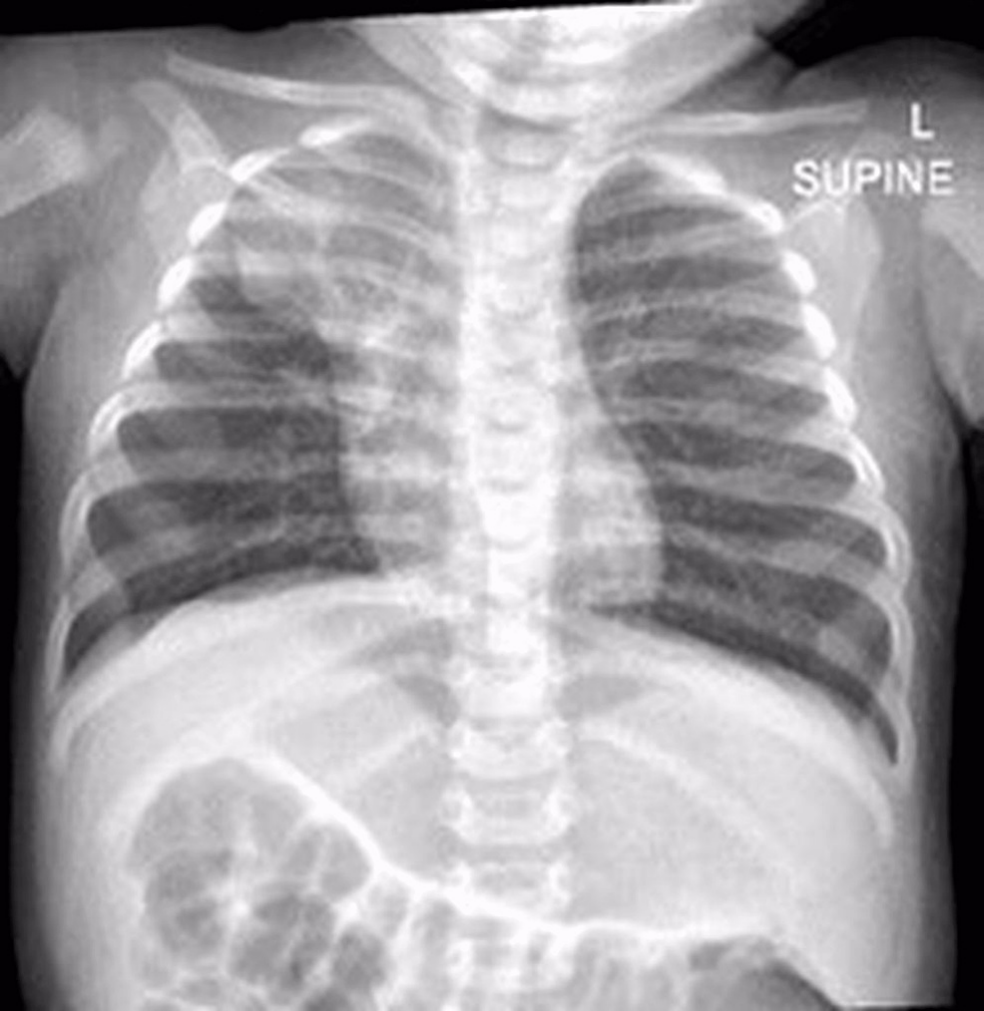
Right upper lobe collapse

Four months later, the patient was admitted to another hospital with fever and respiratory symptoms for two days. After a month, she presented to the emergency department with fever and increased work of breathing; pneumonia was confirmed and treated with HFNC 14 L/min with FiO_2_ of 25%. Intravenous antibiotics were continued for ten days, bronchodilators and a 3% normal saline nebulizer with chest physiotherapy were provided. Chest X-ray showed that the right upper lobe had collapsed (Figure 2). The respiratory culture isolated Streptococcus pneumonia, and the respiratory multiplex was positive for rhino/enterovirus. After completing the antibiotic course, the baby was discharged home. Since then, the baby has done well; she only had to continue chest physiotherapy and hypertonic nebulizer 3%. All her siblings have started to follow up with the pulmonologist after confirming their diagnosis.

## Discussion

Although infants with PCD are often diagnosed with transient tachypnea of the newborn (TTN), the clinical presentation in PCD is different with later onset of respiratory distress, longer duration of oxygen therapy use, and higher frequency of atelectasis and/or lobar collapse upon chest imaging [1].

There is no single gold standard diagnostic test for PCD; the current diagnosis requires a combination of investigations that may not be feasible in all hospitals. Those tests include nasal nitric oxide, high-speed video microscopy analysis, transmission electron microscopy, high-resolution immunofluorescence analysis, and genetic testing [5].

The current therapies for PCD are extrapolated from cystic fibrosis (CF) and patients with non-CF bronchiectasis and lack validation for PCD-specific use [1-6]. The main goal of the treatment is to manage the condition symptomatically, clear the trapped mucus from the airways, and treat the respiratory infection using antibiotics. PCD patients need regular follow-up visits with pediatric pulmonology, otolaryngology, and respiratory therapists. The progression of lung disease varies and is affected by the time of diagnosis, the ability of medical treatment to control symptoms, and the prevention of complications that affect the quality of life [7].

In this case, we reported a full-term neonate with unexplained respiratory distress who needed oxygen therapy for 14 days. Later, her genetic testing was positive for a mutation in the RSPH9 gene, causing PCD. Our patient did not have a laterality defect on chest X-ray and abdominal ultrasound, which made the diagnosis challenging. However, careful history taking of the older siblings, who also had unexplained neonatal respiratory distress and her needing oxygen for a period after birth as well as having chronic wet cough, increased our suspicion about the diagnosis. 

Casey et al. reported similar cases of a family with three individuals who had respiratory distress symptoms as well as PCD, confirmed with genetic testing, associated with laterality defects, which is considered to be due to a homozygous missense variant in CCDC103 on chromosome 17 according to exome sequencing [8]. Another study presented a group of term neonates who had PCD and a history of neonatal respiratory distress [9]. The study showed that cases with PCD required more oxygen therapy for a longer duration and had a later onset of neonatal respiratory distress and a higher frequency of lobar collapse and situs inversus.

It was suggested that when encountering term neonates with unexplained respiratory distress, PCD should be considered in those with lobar collapse, situs inversus, and/or prolonged oxygen therapy (>2 days); moreover, treatment should be initiated immediately to reduce the risk of complications [9]. 

## Conclusions

Primary ciliary dyskinesia (PCD) is a rare autosomal recessive disorder that can present with respiratory distress at birth or in the first few days after birth. Unexplained respiratory distress with the presence of a family history of undiagnosed respiratory symptoms should prompt further investigation for a genetic disease. Whole-exome sequences are the modality of choice for the diagnosis of PCD. The main goal of the treatment is to manage the condition symptomatically, control infections, and clear the trapped mucus from the airways, which can slow the progression of the disease. Careful history taking is vital; in this case, the diagnosis of PCD in three siblings at different ages shows us how PCD is still underdiagnosed and can be easily missed for many years.

## References

[1] Knowles MR, Zariwala M, Leigh M (2016). Primary ciliary dyskinesia. Clin Chest Med.

[2] Machogu E, Gaston B (2021). Respiratory distress in the newborn with primary ciliary dyskinesia. Children.

[3] Meeks M, Bush A (2000). Primary ciliary dyskinesia (PCD). Pediatr Pulmonol.

[4] Hammoudeh S, Gadelhak W, Janahi IA (2019). Primary ciliary dyskinesia among Arabs: where do we go from here?. Paediatr Respir Rev.

[5] Mirra V, Werner C, Santamaria F (2017). Primary ciliary dyskinesia: an update on clinical aspects, genetics, diagnosis, and future treatment strategies. Front Pediatr.

[6] Kido T, Yatera K, Yamasaki K (2012). Two cases of primary ciliary dyskinesia with different responses to macrolide treatment. Intern Med.

[7] Barbato A, Frischer T, Kuehni CE (2009). Primary ciliary dyskinesia: a consensus statement on diagnostic and treatment approaches in children. Eur Respir J.

[8] Casey JP, Goggin P, McDaid J (2015). A case report of primary ciliary dyskinesia, laterality defects and developmental delay caused by the co-existence of a single gene and chromosome disorder. BMC Med Genet.

[9] Mullowney T, Manson D, Kim R, Stephens D, Shah V, Dell S (2014). Primary ciliary dyskinesia and neonatal respiratory distress. Pediatrics.

